# Editorial: Coronavirus Evolution, Cross-Species Transmission and Recombination

**DOI:** 10.3389/fmicb.2021.819417

**Published:** 2022-01-03

**Authors:** Sunil K. Lal

**Affiliations:** School of Science & Tropical Medicine and Biology Multidisciplinary Platform, Monash University Malaysia, Subang Jaya, Malaysia

**Keywords:** SARS-CoV-2, beta coronavirus, ACE-2 receptor, viral transmission, viral infection

We are in the midst of a pandemic where the infective agent has been identified but how it causes mild disease in some and fatally severe disease in other infected individuals still remains a mystery. Two deadly CoV epidemics, Severe Acute Respiratory Syndrome (2003) and Middle East Respiratory Syndrome from the past 20 years already caused enough awareness before the SARS-CoV-2 pandemic emerged in 2019. Looking back, we have been aware of Coronavirus and bats as their reservoir for a very long time but never were we concerned about this more than after the advent of human SARS coronavirus in 2003 and SARS CoV-2 in 2019, resulting in the infamous COVID-19 disease in humans. Labs around the globe have been on an active quest to discover the immediate progenitor to SARS-CoV-2 in bats as well as other intermediate hosts.

Bats are omnipresent on all continents of the world. However, our data on β-CoV transmission is scanty and unevenly distributed globally. We recognize the fact that most reports of transmission to humans occur accidentally through contact with susceptible intermediate animal species, but besides that, there are many other wildlife, natural ecosystems, human activities and social factors that may contribute to viral transmission hot spots of Betacoronavirus (β-CoV) emergence and their detection. This aspect has been studied in detail by Frutos et al. and Hakim et al. in this Research Topic.

According to the virus spillover model for spreading, infected bats are expected to transmit their coronaviruses to an intermediate susceptible host which in turn serves as a source of virus to infect humans. This search for intermediate hosts for the SARS-CoV-2 has led to the discovery of a new set of animals that may have been a part of this transmission cycle. Besides wildlife which certainly had access to contact with bats in the natural ecological environment and may have carried the viral infection to humans in wildlife markets, the possibility of domestic animals as intermediate hosts cannot be ruled out. It becomes important to study the critical role of the intermediate host in the transmission chain of SARS-CoV-2, and the various modes for efficient intervention to stall further cross species transmission of this Coronavirus.

The Malaysian pangolin has emerged as the prime intermediate host candidate that have proved to be carriers of β-CoV that are closely related to SARS-CoV-2 simply due to the fact that they carry the highest number of ACE2 (angiotensin-converting enzyme 2) receptors on their organs with high levels of ACE2 mRNA in their kidneys. This aspect has been well-studied for captive pangolins in the Guangdong province of China, by Li et al. in this Research Topic.

This study clearly brings to light the importance of monitoring and understanding the coronavirus spectrum carried by animals over extended periods of time, especially pangolins and cataloging them for future identification and tracing.

*In silico* modeling of the pangolin ACE2 receptor has suggested that the SARS-CoV-2 and its emerging variants could have altered affinity of the spike protein from a pangolin coronavirus which may bind with increased affinity to both pangolin and Homo sapiens ACE2 (Hsap ACE2) receptors. Similarly, investigations on ACE2 polymorphism indicate that the list of SARS-CoV-2 susceptible species may also include the Macaca mulatta (monkey), Felis catus (cat), Canis lupus (dog), Oryctolagus cuniculus (rabbit), Mustela putorius furo (ferret), Mesocricetus auratus (hamster), Bos taurus (cow), Bubalus bubalus (buffalo), Capra hircus (goat), Ovis aries (sheep), and the Mustelidae family (minks), all found to be susceptible to SARS-CoV-2 infection and the virus was efficiently transmitted from one infected animal to another via respiratory droplets. This Research Topic collection, deals with a similar study by Devaux et al. on minks as an excellent disease model for studying the human and animal interface by examining SARS-CoV-2 infection of minks in farms to analyze selective sweeps of the virus and its variants that pass from Mustelidae to humans. Also in this context it becomes important to assess the risk of mink evolved SARS-CoV-2 variants in humans and their response to current vaccines.

At this time, it becomes important for us to understand the huge complexity of over 39,000 variants and increased polymorphisms exhibited by SARS-CoV-2 which increases the diversity and chance variation prospects of this virus. Farkas et al. have developed a computational pipeline to assist researchers in the rapid analysis and characterization of these SARS-CoV-2 variants and their accurate identification.

Our collection of carefully selected articles in this Research Topic by experts in this field make up an important compilation of relevant articles primarily focussing on the evolutionary and cross-species transmission aspects of Coronavirus.

## Author Contributions

The author confirms being the sole contributor of this work and has approved it for publication.

## Conflict of Interest

The author declares that the research was conducted in the absence of any commercial or financial relationships that could be construed as a potential conflict of interest.

## Publisher's Note

All claims expressed in this article are solely those of the authors and do not necessarily represent those of their affiliated organizations, or those of the publisher, the editors and the reviewers. Any product that may be evaluated in this article, or claim that may be made by its manufacturer, is not guaranteed or endorsed by the publisher.

